# Total ankle arthroplasty and ankle arthrodesis affect the biomechanics of the inner foot differently

**DOI:** 10.1038/s41598-019-50091-6

**Published:** 2019-09-16

**Authors:** Yan Wang, Duo Wai-chi Wong, Qitao Tan, Zengyong Li, Ming Zhang

**Affiliations:** 1 Department of Biomedical Engineering, Faculty of Engineering, The Hong Kong Polytechnic University, Kowloon, Hong Kong, China; 2 The Hong Kong Polytechnic University Shenzhen Research Institute, Shenzhen, China; 3 The National Research Center for Rehabilitation Technical Aids, Beijing, China

**Keywords:** Skeleton, Biomedical engineering

## Abstract

Ankle arthrodesis and total ankle arthroplasty are the two primary surgeries for treatment of end-stage degenerative ankle arthritis. The biomechanical effects of them on the inner foot are insufficient to identify which is superior. This study compared biomechanical parameters among a foot treated by ankle arthrodesis, a foot treated by total ankle arthroplasty, and an intact foot using computational analysis. Validated finite element models of the three feet were developed and used to simulate the stance phase of gait. The results showed total ankle arthroplasty provides a more stable plantar pressure distribution than ankle arthrodesis. The highest contact pressure, 3.17 MPa, occurred in the medial cuneonavicular joint in the total ankle arthroplasty foot. Neither of the surgeries resulted in contact pressure increase in the subtalar joint. The peak stress in the metatarsal bones was increased in both surgical models, especially the second and third metatarsals. This study enables us to get visual to the biomechanics inside of an intact foot, and feet treated by total ankle arthroplasty and ankle arthrodesis during walking.

## Introduction

Ankle arthrodesis and total ankle arthroplasty (TAA) are the two primary surgeries for treatment of end-stage degenerative ankle arthritis. Despite promising results for most patients, both surgeries lead to various complications. Ankle arthrodesis, considered as the gold standard of treatments, eliminates ankle joint motion, potentially resulting in bony fracture, malalignment, nonunion, adjacent joint degeneration, and foot pain^1–5^. TAA has been advocated as an alternative because it preserves ankle joint motion^6,7^, but complications such as fracture, implant loosening, and malalignment often arise^8–11^. Studies of gait analysis^12–14^, cadaveric experiments^15,16^, physical tests, radiological examination, and pain/function scores^17,18^ have compared the two surgical treatments regarding the functional outcomes and complications. Due to the difficulty of imaging the inside of the foot, the biomechanical deviations of the inner foot are seldom revealed^19–21^.

Computational models of the human foot and ankle have been used to explore the biomechanics of surgery. Three-dimensional finite element (FE) models of ankles were developed to compare the stability^22,23^ and stress in bones and implants^23^ of different techniques in ankle arthrodesis surgery. To evaluate the biomechanical environment of the bone and intramedullary nail in ankle arthrodesis surgery, a FE model of ankle bones was developed and used to analyze the stress in bones and nails^24^. FE models of TAA were developed to understand the contact pressure and implant kinematics of the implants^25^, the alignment of prosthetic components^26^, the failure mechanism of the polyethylene component^27^, the process of bone remodeling after TAA^28^, and plantar pressure and bone stress distribution^29^. These investigations provided valuable insights into the biomechanical consequences of TAA and ankle arthrodesis, but separately, making it difficult to conduct a direct comparison between the two surgeries. These models were also designed to represent the regions directly operated on, making it impossible to evaluate the effects of these surgeries on the entire foot, even the adjacent areas. Models with more detailed anatomical representations of the foot would be capable of simulating more sophisticated behaviors. This study aimed to compare the biomechanical effects of TAA and ankle arthrodesis on the foot using a FE model that represented most of the anatomical structures of the foot and ankle. Models of an intact foot, a foot with TAA, and a foot with ankle arthrodesis were used to simulate the stance phase of gait, and a comparison of biomechanical parameters among the three models was conducted. It is found that forces transferred through foot segments were deviated in the foot treated by total ankle arthroplasty and the foot treated by ankle arthrodesis from an intact foot, resulting in changes of joint contact pressure, bone stress, plantar pressure distribution and foot deformation during gait.

## Results

The biomechanical performances of the foot and ankle during gait were analyzed using the validated FE models. Biomechanical parameters including plantar pressure, joint contact pressure, joint contact force, stress distribution in metatarsals, and foot displacement were compared among the intact foot, TAA foot, and ankle arthrodesis foot at the first-peak, mid-stance, and second-peak instants during gait.

### Model validation

The plantar pressure distributions were compared between the FE prediction and F-Scan measurements under three conditions including balanced standing, the first-peak, and second-peak instants. In balanced standing, the average peak pressure in the fore- and hind-foot was 0.051 MPa and 0.168 MPa in the FE prediction and 0.058 MPa and 0.157 MPa in the experimental measurements. In the hind-foot at the first-peak instant, these values were 0.3 MPa and 0.307 MPa, respectively, in the FE prediction and experimental measurements. The averaged plantar pressure in the fore-foot at the second-peak instant were 0.227 MPa and 0.223 MPa, respectively, in the FE prediction and experimental measurements. To compare the contact pressure of the navicular joint between calculation using the FE model and measurement in the cadaveric experiment, the FE model and the cadaver foot were subjected to the same boundary and loading conditions. The averaged pressure was 0.25 MPa in the FE prediction, and the physical measurement was 0.26 MPa in the cadaveric experiment. These comparisons showed a satisfactory agreement between the model predictions and experimental measurements.

### Plantar pressure

**Figure 1:**
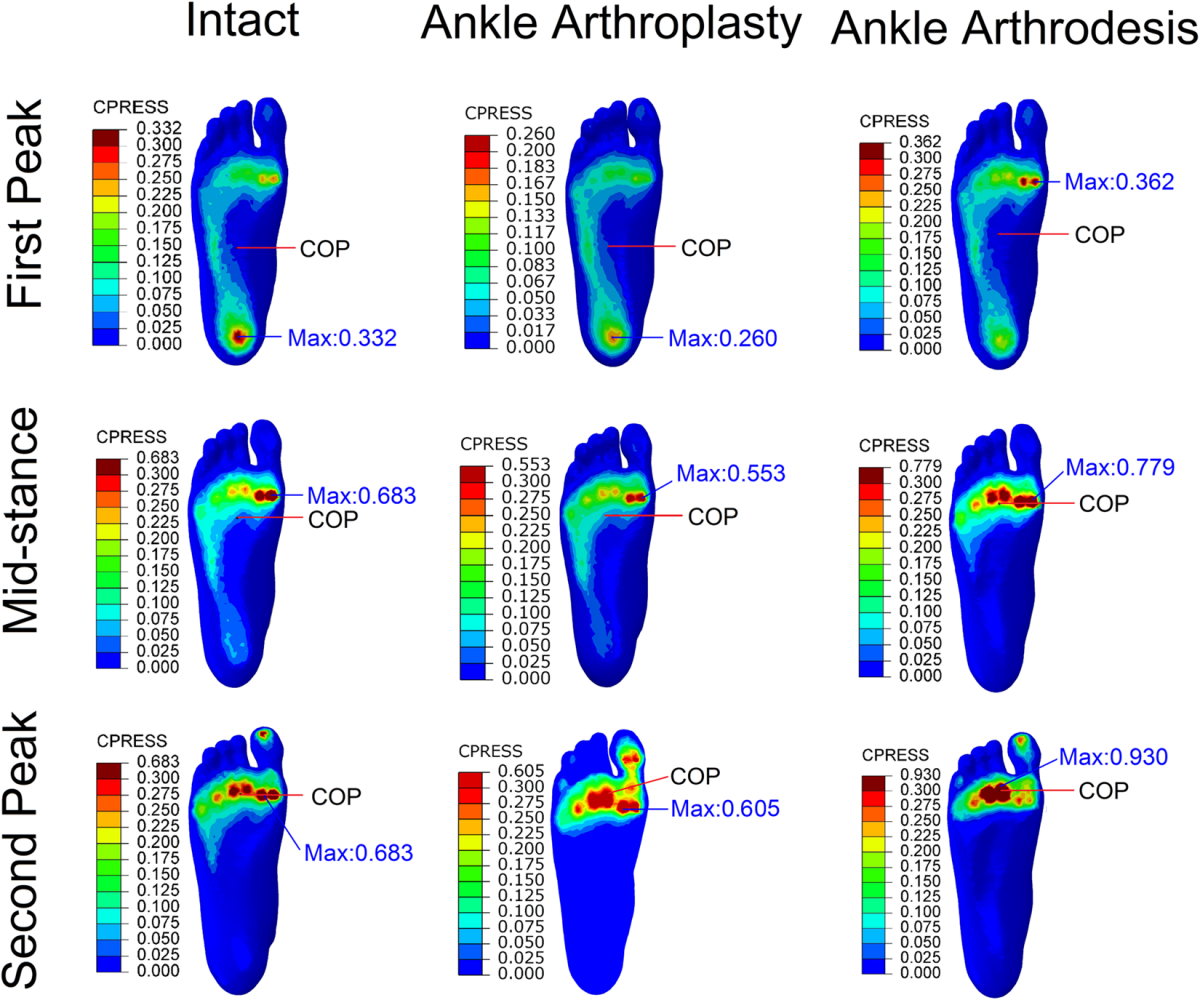
Comparison of plantar pressure distributions and the location of center of pressure (COP) among the intact foot model, total ankle arthroplasty model, and ankle arthrodesis model at the first-peak, mid-stance, and second-peak instants.

The plantar pressure distributions in the three foot models at the three instants are shown in Fig. 1. In the model of the intact foot, the peak pressure was 0.332 MPa, 0.683 MPa, and 0.683 MPa, respectively, at the first-peak, mid-stance, and second-peak instants, while that in the TAA foot model was 0.26 MPa, 0.553 MPa, and 0.605 MPa and that in the ankle arthrodesis foot was 0.362 MPa, 0.779 MPa, and 0.930 MPa, i.e. 9%, 14%, and 36% higher than in the intact foot model. It was noted that, relative to the intact foot, the ankle arthrodesis foot had more deviation in the peak plantar pressure than did the TAA foot. The location of the center of pressure in the TAA foot was not different from that in the intact foot, while in the ankle arthrodesis foot it was anteriorly shifted by 15 mm, 16 mm, and 5 mm at the three instants, respectively, compared with the intact foot.

### Joint contact pressure

**Figure 2:**
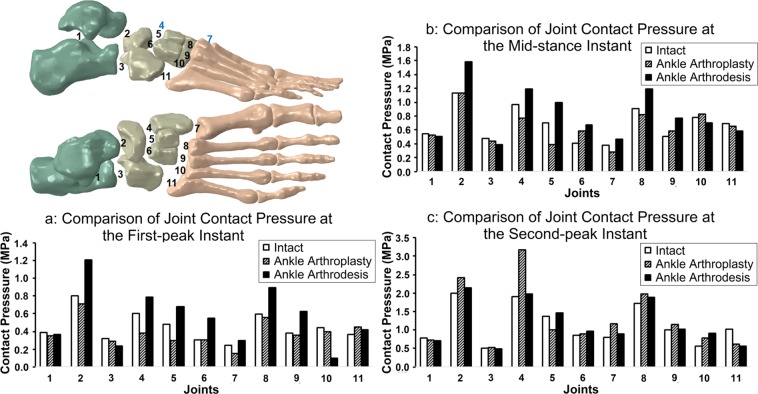
Comparison of joint contact pressure in the intact foot model, total ankle arthroplasty foot model, and ankle arthrodesis foot model at the first-peak, mid-stance, and second-peak instants, including (**a**) comparison of joint contact pressure among the three models at the first-peak instant, (**b**) comparison of joint contact pressure among the three models at the mid-stance instant, and (**c**) comparison of joint contact pressure among the three models at the second-peak instant. Joints: 1 - Subtalar, 2 - Talonavicular, 3 - Calcaneocuboid, 4 - Medial cuneonavicular, 5 - Intermediate cuneonavicular, 6 - Lateral cuneonavicular, 7 - First tarsometatarsal, 8 - Second tarsometatarsal, 9 - Third tarsometatarsal, 10 - Fourth tarsometatarsal, 11 - Fifth tarsometatarsal.

Figure 2 shows the contact pressures at 11 joints. In most joints, the contact pressure for both surgical models deviated markedly from the intact foot model, while it remained stable in the subtalar joint.

At the first-peak instant (Fig. 2a), the contact pressure was decreased at the subtalar joint (1), calcaneocuboid joint (3), and fourth tarsometatarsal joint (10) in both surgical foot models compared with that in the intact foot model. Comparing the TAA and the intact foot model, the contact pressure in 10 out of the 11 joints was decreased in the former, the only exception being a 21.7% increase in the fifth tarsometatarsal joint. Comparing the ankle arthrodesis and the intact foot model, the contact pressure was increased in the former at eight out of the 11 joints, including the talonavicular (2), medial cuneonavicular (4), intermediate cuneonavicular (5), lateral cuneonavicular (6), first tarsometatarsal (7), second tarsometatarsal (8), third tarsometatarsal (9), and fifth tarsometatarsal (11), by 50.5%, 31.1%, 41.6%, 80.3%, 21.9%, 49.7%, 62.8%, and 13.8%, respectively. At the first-peak instant, the highest contact pressure among all models occurred at the talonavicular joint, which was 1.21 MPa, in the ankle arthrodesis foot model.

At the mid-stance instant (Fig. 2b), the contact pressure was decreased at the subtalar joint (1), calcaneocuboid joint (3), and fifth tarsometatarsal joint (11) in both surgical foot models compared with the intact foot model. Comparing the TAA and the intact foot model, the contact pressure in eight out of the 11 joints was decreased in the former, except for 44.3%, 15.1%, and 6.3% increases, respectively, at the lateral cuneonavicular (6), third tarsometatarsal (9), and fourth tarsometatarsal (10). Comparing the ankle arthrodesis and the intact foot model, the contact pressure at seven joints was increased in the former, including the talonavicular (2), medial cuneonavicular (4), intermediate cuneonavicular (5), lateral cuneonavicular (6), first tarsometatarsal (7), second tarsometatarsal (8), and third tarsometatarsal (9), by 39.6%, 23.9%, 41.8%, 64.7%, 24.4%, 30.9%, and 51.4%, respectively. At the mid-stance instant, the highest contact pressure among all models occurred at the talonavicular joint, which was 1.59 MPa, in the ankle arthrodesis foot model.

At the second-peak instant (Fig. 2c), the contact pressure was decreased at the subtalar (1) and fifth tarsometatarsal (11) joints in both surgical foot models compared with the intact foot model. Comparing the TAA and the intact foot model, the contact pressure at the subtalar (1), intermediate cuneonavicular (5), and the fifth tarsometatarsal (11) joints was decreased in the former. That at 8 out of the 11 joints was increased, including the talonavicular (2), calcaneocuboid (3), medial cuneonavicular (4), lateral cuneonavicular (6), first tarsometatarsal (7), second tarsometatarsal (8), third tarsometatarsal (9), and fourth tarsometatarsal (10), by 20.5%, 4.1%, 67.4%, 3.6%, 44.0%, 15.6%, 14.7%, and 37.5%, respectively. Comparing the ankle arthrodesis and the intact foot model, the contact pressure increased in the former at eight joints, including the talonavicular (2), medial cuneonavicular (4), intermediate cuneonavicular (5), lateral cuneonavicular (6), first tarsometatarsal (7), second tarsometatarsal (8), third tarsometatarsal (9), and fourth tarsometatarsal (10), by 6.8%, 3.9%, 7.3%, 11.6%, 11.6%, 9.7%, 2.0%, and 63.1%, respectively. At the second-peak instant, the highest contact pressure among all models occurred at the medial cuneonavicular joint (4) in the TAA foot model, which was 3.17 MPa.

### Force transfer in foot segments

**Figure 3:**
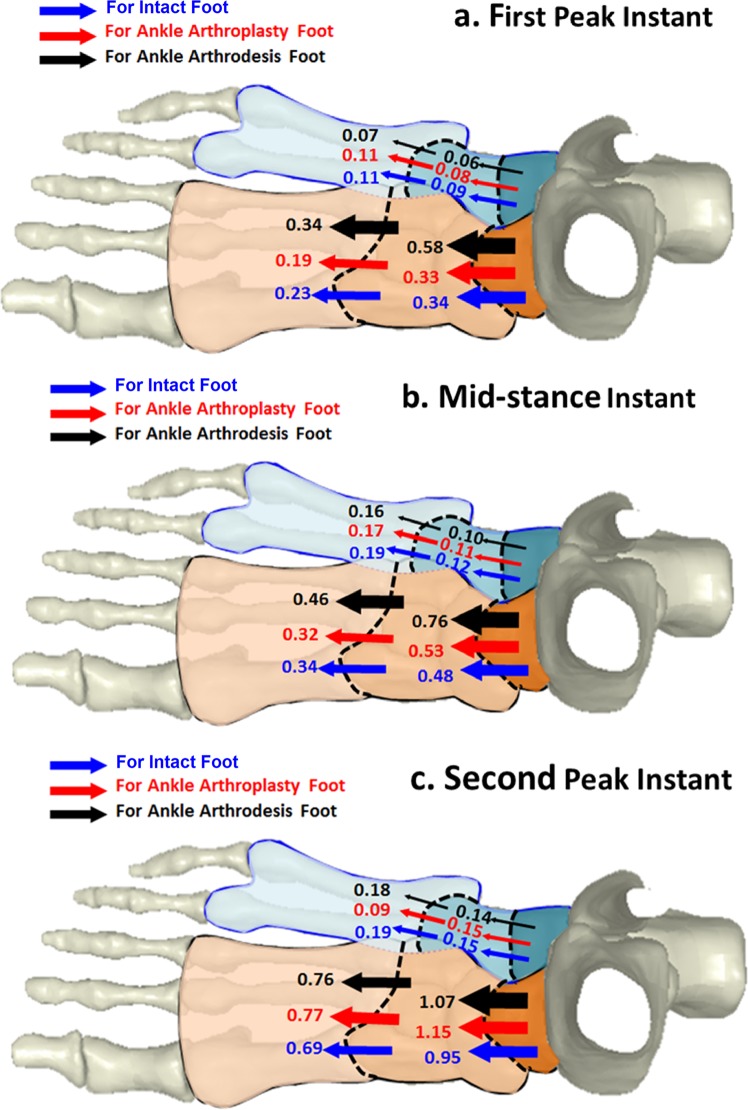
Comparison of force distribution in foot segments among intact foot model, total ankle arthroplasty model, and ankle arthrodesis model: (**a**) force distribution in three foot models at the first-peak instant; (**b**) force distribution in three models at the mid-stance instant; and (**c**) force distribution in three foot models at the second-peak instant. Forces transferred through joints are represented in multiples of body weight.

A comparison of force transfer distribution, presented as multiples of body weight, was conducted among the three models at the three instants. Figure 3a depicts the force distribution in the three foot models at the first-peak instant. Force was transferred from the hind-foot to the mid-foot through the talonavicular and calcaneocuboid joints. Forces of 0.34, 0.33, and 0.58 times body weight were transferred through the talonavicular joint in the intact foot model, TAA model, and ankle arthrodesis^30^ model, respectively. At the same time, forces of 0.09, 0.08, and 0.06 times body weight were transferred through the calcaneocuboid joint, respectively, in the three models.

Forces were delivered from the mid-foot to the fore-foot through the five tarsometatarsal joints. From medial to lateral side, the first to third metatarsals were set as the medial pathway, and the fourth to fifth metatarsal were set as the lateral pathway, as shown in Fig. 3. The medial aspect sustained 0.23, 0.19, and 0.34 times body weight, respectively, in the three models, while 0.11, 0.11, and 0.07 times body weight were transferred through the lateral side.

Figure 3b shows the force distribution in the three models at the mid-stance instant. From the hind-foot to the mid-foot, the talonavicular joint undertook forces of 0.48, 0.53, and 0.76 times body weight, while the calcaneocuboid joint sustained 0.12, 0.11, and 0.10 times body weight, respectively, in the three models. At the articulations between the mid- and fore-foot, the medial aspect delivered forces of 0.34, 0.32, and 0.46 times body weight, respectively, in the three models, and 0.19, 0.17, and 0.16 times body weight were transferred through the lateral side.

Figure 3c depicts the force distribution at the second-peak instant. At the articulations between the hind- and mid-foot, the talonavicular joint sustained forces of 0.95, 1.15, and 1.07 times body weight, respectively, in the three models, and the calcaneocuboid joint undertook 0.15, 0.15, and 0.14 times body weight loading, respectively. At the articulation between the mid- and fore-foot, the medial aspect delivered forces of 0.69, 0.77, and 0.76 times body weight, respectively, in the three models, and the lateral aspect of the metatarsals undertook 0.19, 0.09, and 0.18 times body weight loading, respectively, in the intact foot model, TAA foot model, and ankle arthrodesis foot model.

### Bone stress

**Figure 4:**
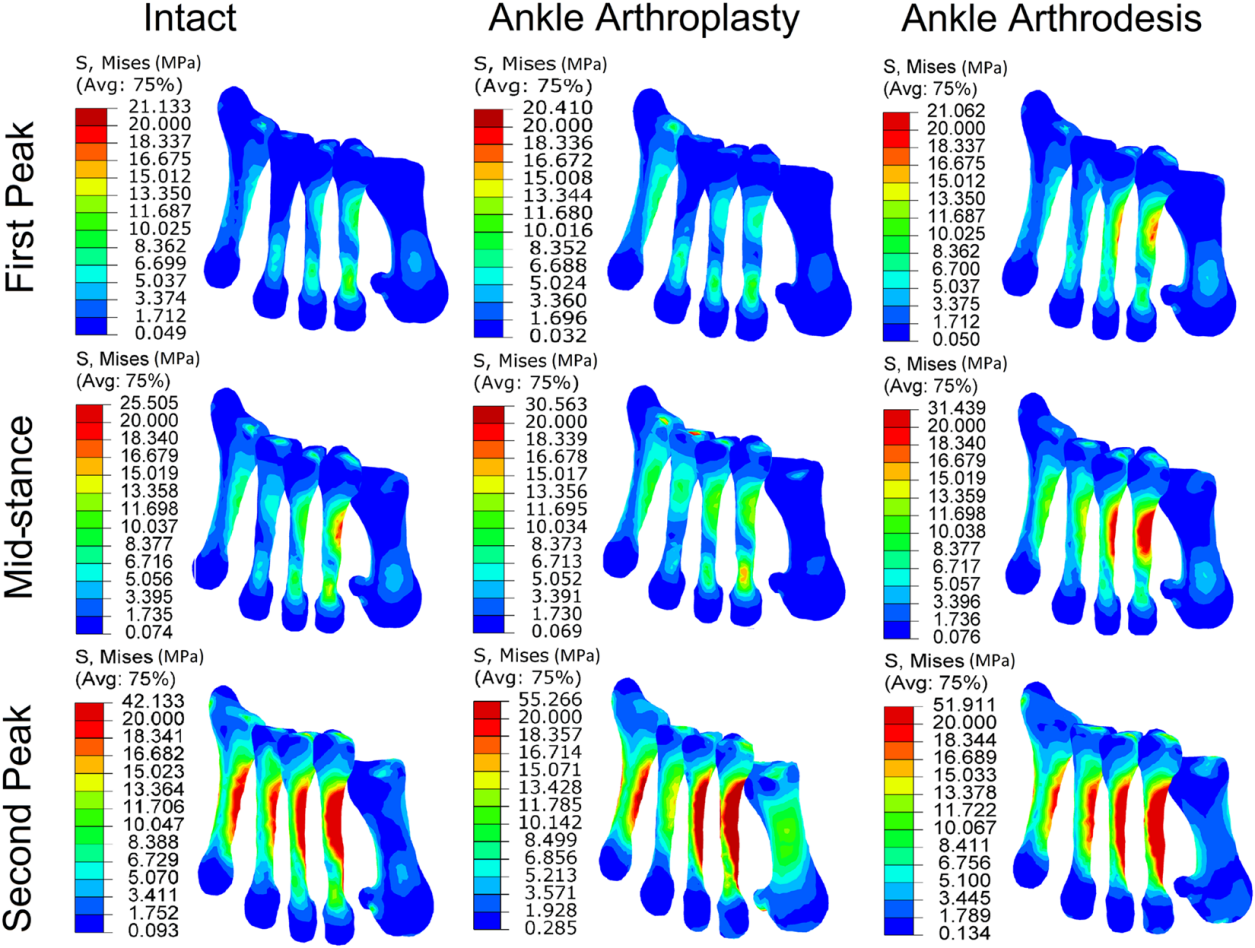
Comparison of stress distribution in the intact foot model, total ankle arthroplasty model, and ankle arthrodesis model at the first-peak, the mid-stance, and the second-peak instants.

The stress distributions in the metatarsal bones are compared in Fig. 4. At the first-peak instant, the peak stress was 21.1 MPa, 20.4 MPa, and 21.1 MPa, respectively, in the intact foot model, the TAA model, and the ankle arthrodesis model. Compared with the intact foot model, the peak stress in the TAA model was 3.32% lower, while that in the ankle arthrodesis model was almost unchanged. At the mid-stance instant, the peak stress was 25.5 MPa, 30.6 MPa, and 31.4 MPa, respectively, in the three foot models. Compared with the intact foot model, the peak stress was 20% higher in the TAA model and 23.1% higher in the ankle arthrodesis model. At the second-peak instant, the peak stress was 42.1 MPa, 55.3 MPa, and 51.9 MPa, respectively, in the three models. Compared with the intact foot model, it was 31.2% higher in the TAA model and 23.2% higher in the ankle arthrodesis model.

### Foot displacement

**Figure 5:**
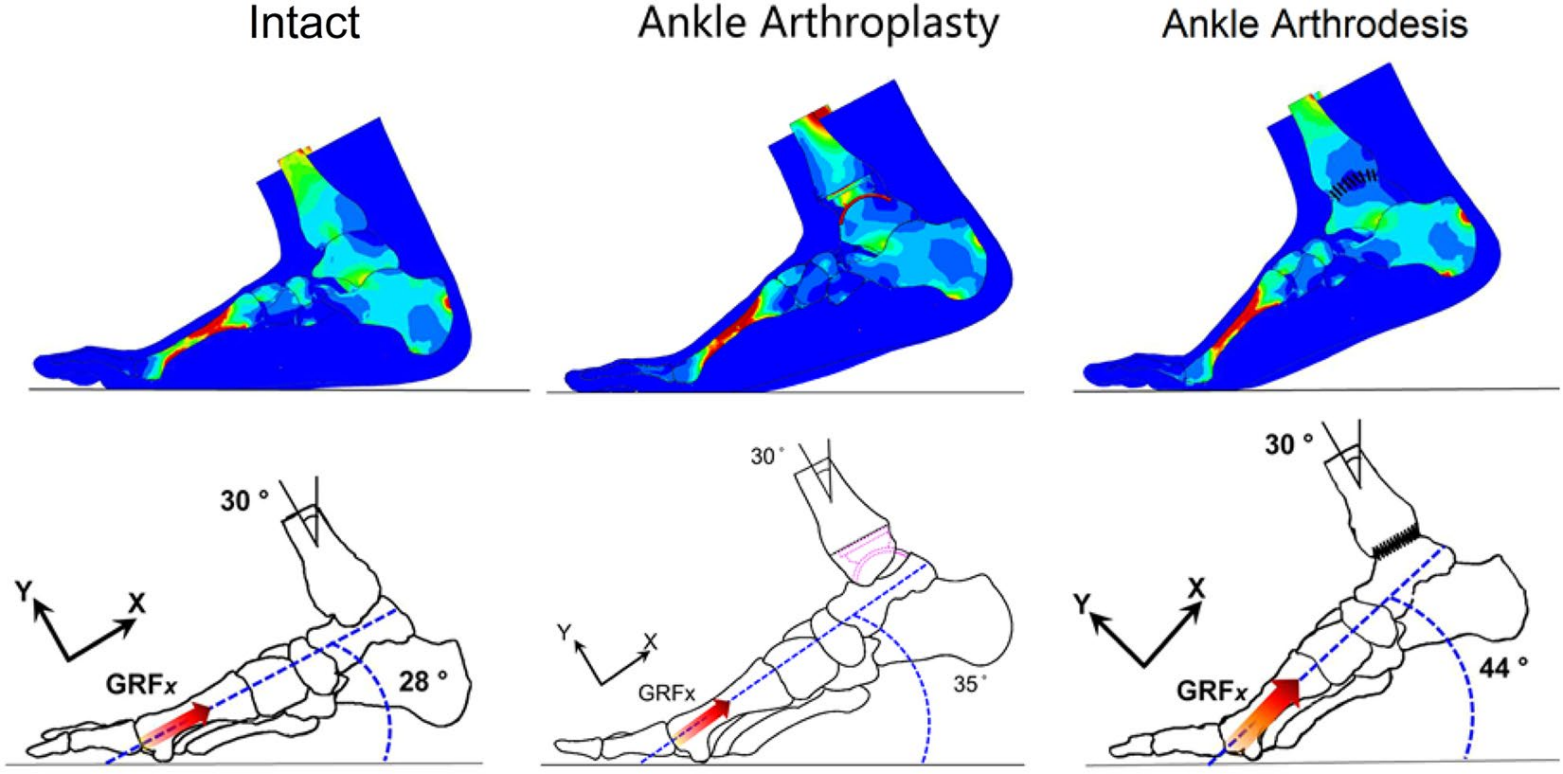
Comparison of foot displacement among the intact foot model, total ankle arthroplasty model, and ankle arthrodesis model at the second-peak instant.

The TAA foot, the ankle arthrodesis foot, and the intact foot showed comparable angular displacement in the fore-foot at the first-peak and mid-stance instants, while the displacements at the second-peak instant differed among the three models, as shown in Fig. 5. The position of the foot shank was the same in all three models, with a 30° deviation from the vertical direction. The angle between the ground and the axis along the first ray (the medial cuneiform, the first metatarsal, and the first phalange) was 28° in the intact foot model, 35° in the TAA model, and 44° in the ankle arthrodesis model.

## Discussion

As a versatile biomechanical structure, the foot and ankle complex provides body support, propulsion, and impact absorption. Surgeries change the performance of this complex, with resultant deviation in the kinematics and kinetics of the inner foot and the plantar foot loading. In this study, the biomechanical consequences of total ankle arthroplasty and ankle arthrodesis were investigated and compared in terms of joint contact pressure, plantar pressure, bone stress distribution, and force transmission.

Plantar pressure has indicated relevance as an outcome marker in clinical application^31^, and has been used to enhance understanding of foot function and effectiveness of therapeutic and surgical interventions in treatment of foot disorders^32^. The prediction based on the results in this study was supportive of earlier findings that TAA did not increase the peak pressure nor change the location of the center of pressure^33^, while ankle arthrodesis increased the peak pressure^34^ and anteriorly advanced the center of pressure. At the first-peak and mid-stance instants, the TAA foot permitted a comparable ankle joint motion to the intact foot, while the ankle arthrodesis foot, with totally constrained ankle, required additional displacement of the fore-foot to compensate the ankle dorsiflexion and forward tilt of the tibia bone during gait progression^35,36^. Such a compensation can drive earlier heel-off^35^ and a more anterior center of pressure than in an intact foot, as predicted in this study. The peak pressure in the ankle arthrodesis foot therefore occurred in the fore-foot beneath the head of the first metatarsal, rather than in the heel region as in the intact foot and TAA foot. Increased plantar pressure could potentially result in discomfort during weight-bearing activities, and might be a risk factor for plantar foot pain after surgery. Patients tend to adapt to the variability and minimize adverse effects by adjustment in foot posture, walking speed, and cadence postoperatively^37^.

At the first-peak and mid-stance instants, the joint contact pressure in the TAA foot was close to that of the intact foot, but substantial variation occurred around the second-peak instant. Ankle arthrodesis resulted in more marked effects at the first half of the stance phase, but provided relatively stable biomechanics at the second-peak instant. The contact pressure at the talonavicular and cuneonavicular joints in the ankle arthrodesis foot at the first-peak and mid-stance instants was much higher than that in the intact foot and the TAA foot. Increased contact pressure is indicative of higher risk of degenerative changes at affected joints after repetitive loading cycles. Studies have demonstrated arthrosis at joints including talonavicular, cuneonavicular, and tarsometatarsals following ankle arthrodesis^34^, which was consistent with the prediction in this study. The finding that neither TAA nor ankle arthrodesis resulted in an increase of joint contact pressure at the subtalar joint strengthened the notion that arthrosis in the subtalar is more likely to be a progression of preexisting degeneration than a consequence of ankle arthrodesis^38^.

The most substantial variations of inner foot loadings occurred in the TAA foot at the second-peak instant, which greatly exceeded the level of the intact foot. Increased loadings could be deteriorative factors of postoperative complications under repeated gait cycles and other weight-bearing activities. In some studies, TAA was demonstrated to give rise to more complications and need for secondary surgical intervention than ankle arthrodesis^39,40^.

The range of ankle joint motion in the TAA and ankle arthrodesis models was smaller than in the intact foot, with the smallest occurring in the ankle arthrodesis foot. This is because three-component ankle prostheses can preserve part of the ankle joint motion while ankle arthrodesis totally constrains the motion at the ankle joint^33,41–43^. This finding was consistent with kinematic studies on TAA^41,44^ and ankle arthrodesis feet^35,45,46^. The range of motion of the fore-foot in both surgical foot models was larger than that in the intact foot, with the largest occurring in the ankle arthrodesis foot. In ankle arthrodesis feet, tibial tilt relative to the ground is enabled by the early heel lift^35,47^, resulting in larger deformation in the fore-foot, as predicted in this study. This is likely to represent a compensatory mechanism for the limited capacity of the ankle joint. Existing designs of three-component ankle joints theoretically allow the full range of dorsi- and plantarflexion in the sagittal plane but totally constrain eversion/inversion motion in the frontal plane. However, the oblique axis of the ankle joint motion indicates complexed motion in the three planes^48^. The progression of the gait depends on the synergistic effects of joints in the lower limb, especially in the foot and ankle. To maintain the function of the foot and ankle complex, each unit adjusts slightly in the most efficient way to adapt to deviations in specific position. The limited range of motion in the sagittal plane is possibly a consequence of the constrained eversion/inversion.

The results were expected to predict how the biomechanics of the foot, particular the inner foot, were differently affected by total ankle arthroplasty and ankle arthrodesis surgeries, rather than representing the exact data of the inner foot in real cases in consideration of the limitations in the study. First, the computational models were based on simplifications and assumptions. The bones in the FE model were reconstructed without separation of cortical and trabecular components and were assigned as homogeneous, isotropic, and linear elastic material. Second, the boundary and loading conditions applied to the ankle arthrodesis foot and TAA foot were the same as that of the intact foot. For ease of comparison it was assumed that patients after surgical treatment would keep the same gait pattern, although some patients may in fact adjust their walking pattern for comfort. Further studies should include motion analysis of ankle arthrodesis and TAA patients, to improve the reasonability of the results. Third, the force and pressure transmission was by the immediate contact among solid bodies of the bones, which can be improved by application of the new finding on pressure transmission between synovial capsules via subperiosteal space^49^. Although the joint contact behavior was represented using a cartilage contact curve applied to the contact surface of bones, it can be more reasonable by development of the geometries of the cartilage for application of cartilage mechanisms^50^. For a more practical investigation of the biomechanics of the inner foot in future studies, the computational models will be modified to involve the mechanism of subperiosteal transmission of pressure and the cartilage contact mechanism, The development of the cartilage geometries in the FE model will possibly expand the way of application of boundary conditions and strengthen the validation of the model using the contact area and deformation in the cartilage^51^. Furthermore, the models were constructed based on one female subject attempting to represent normal functioned foot behaviors, individual differences were thus not involved. For example, the difference in foot size, body weight, or gait pattern may result in variation of the specific values of the inner foot biomechanics. The STAR ankle prosthesis has five sizes to fit different foot sizes, and in this study only the extra small size was adopted. Depending on the experience and the specific characteristics of the subject symptoms, in ankle arthrodesis surgeons sometimes fuse the ankle joint in a slightly valgus position. However, based on the debates that fusing the ankle in a neutral position allows the use of any remaining mid-foot motion, which could compensate for some ankle joint motion and lead to desired outcomes^35,52–54^. Thus, the ankle joint was fused in the neutral position in the FE model. Currently, model development protocols of foot models of TAA or ankle arthrodesis are generally the same as in this study that the foot model was reconstructed from medical images of a normal function foot, and based on which the ankle joint was replaced by ankle prosthesis or fused by screws in neutral position^22,23,28^. Foot models based on the relevant pathological foot^29^ could be more reasonable than normal-foot-based model for comparison of biomechanics before and after surgeries. The main differences of foot models among existing studies are the inclusion of partial or complete foot-ankle structures, and consequent difference in applications of boundary and loading conditions. Comparing models of ankle arthrodesis, fixation screws were developed in studies that focused on the effectiveness of fixation approaches, while this study, focusing on the biomechanics of the entire foot, using ‘tie’ connection to represent screw fixation. All these individual and operational differences may lead to variation of the specific values, thus this study is expected to demonstrate the variation trend of force transmission after TAA and ankle arthrodesis surgeries rather than exact representation of individual cases. As this study simulated three characteristic instants in the stance phase of the gait, plantar pressure at three conditions was measured and compared to the simulation for validation. In further study, simulation will be conducted to simulate the continuous walking process to provide more sufficient data than three instants, and the validation would be through comparison of data over one stride period rather than three instants.

## Conclusion

Neither TAA nor ankle fusion totally preserved the anatomical characteristics of a natural ankle joint, hence resulting in deviation in ankle joint motion, which was compensated by the angular displacement in the fore-foot. This had consequences for the force transmission among segments, joint contact pressure, and bone stress distribution. Comparing these parameters among the intact foot model, and the two surgical models of TAA ankle arthrodesis, TAA induced a more acceptable plantar pressure and ankle motion in the sagittal plane than ankle arthrodesis. In terms of joint contact pressure, the most substantial variation and the highest pressure occurred in the TAA foot at the medial cuneonavicular joint. Neither surgery resulted in any increase of contact pressure in the subtalar joint. The information obtained in this study might be used to predict and compare surgical outcomes.

## Methods

Ethical approval for this project was granted by The Hong Kong Polytechnic University Human Subject Ethics Committee (reference number HSEARS20070115001)^30^, and all research was performed in accordance with relevant guidelines and regulations. The subject was informed of the experimental procedures and gave written informed consent for participating in the magnetic resonance scanning and gait measurements.

**Figure 6:**
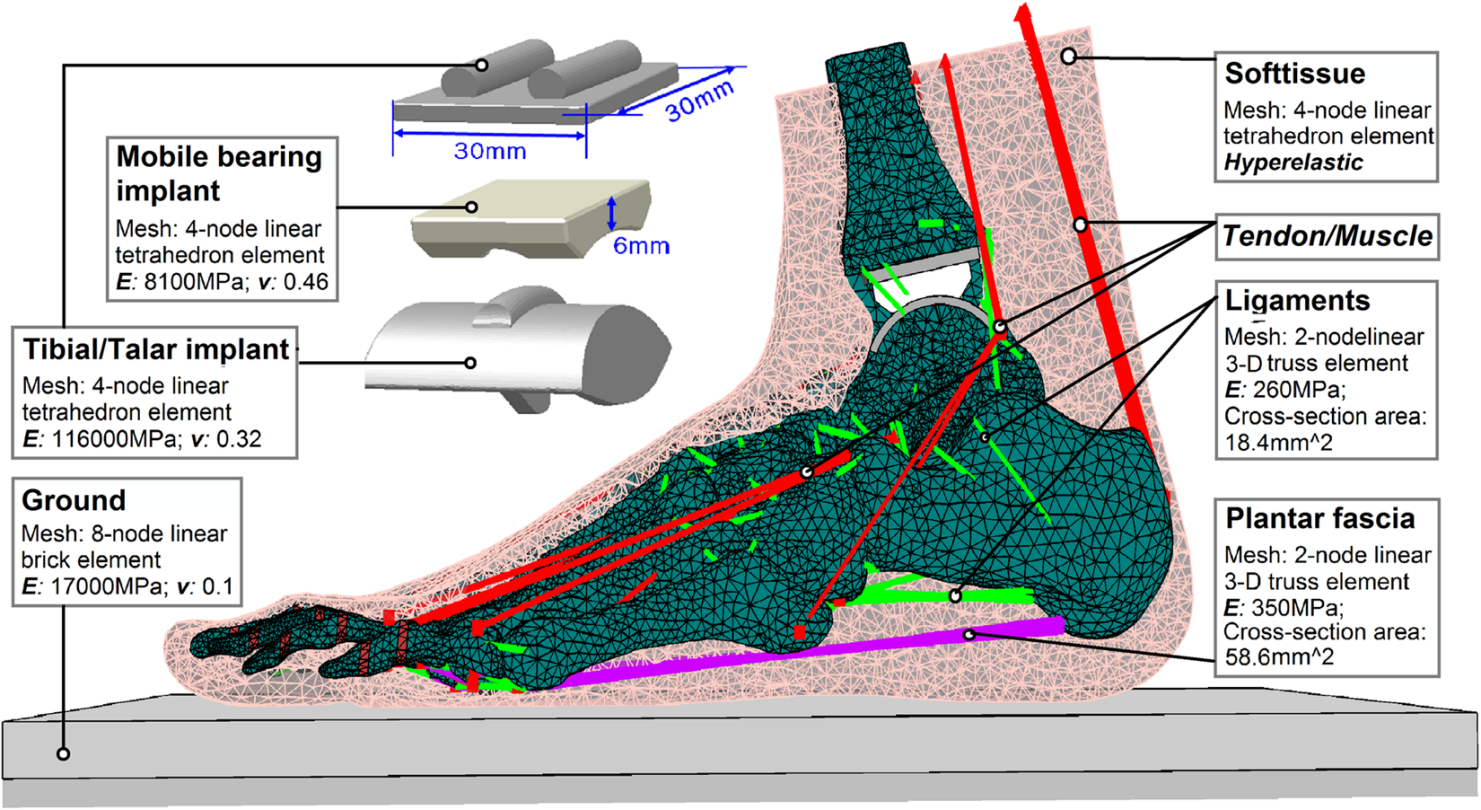
Finite element model of the foot and ankle with total ankle arthroplasty, and parameters of material properties and mesh.

A FE model of an intact foot and ankle complex was developed by reconstruction of magnetic resonance images (resolution of 0.625 mm, interval of 2 mm) of the right foot of a female subject with body height of 164 cm and body mass of 54 kg^30^. The subject had no history of injuries or pathologies in the musculoskeletal system of the lower limbs. The geometries of 28 bones and a bulk of encapsulated soft tissue were reconstructed in MIMICS (Materialise, Leuven, Belgium). These bones and soft tissue were assembled into an integrated foot-ankle structure, as shown in Fig. 6, in the FE software package ABAQUS (Dassault Systèms Simulia Corp., Providence, RI, USA). The plantar fascia and 103 ligaments were simulated with tension-only wire elements connecting at the insertion points of corresponding bones. Nine groups of extrinsic muscles were included, namely the Achilles tendon merged with the triceps surae, the tibialis anterior, tibialis posterior, peroneus longus, peroneus brevis, flexor hallucis longus, flexor digitorum longus, extensor hallucis longus, and extensor digitorum longus. Each group of muscles was constructed by connecting the attachment points on the bones using an axial connector, which allows application of concentrated muscle forces. The joint interfaces of bones were set as frictionless surface-to-surface contact, and non-linear contact stiffness was applied to the joint contact surface to represent the cartilaginous layers. Two layers of plates were constructed and tied together beneath the plantar foot, with the upper layer representing a concrete ground and the lower layer set as a rigid body for the application of ground reaction forces. The ground plate was assigned the properties of concrete, with the Young’s Modulus (***E***) and Poisson’s ratio (***v***) of 17000 MPa and 0.1, respectively. The bones (***E*** 7300 MPa, ***v*** 0.3), ligaments (***E*** 260 MPa, cross-section area 0.3 mm^2^), and plantar fascia (***E*** 350 MPa, cross-section area 58.6 mm^2^) were assumed to be homogeneous, isotropic, and linearly elastic materials, while the bulk of soft tissue was modeled with non-linear hyperelasticity. The hyperelastic behavior was represented by a second-order polynomial strain energy potential expression (Eq. 1) (ABAQUS User’s Manual) in the form of

1 $${\boldsymbol{U}}={\sum }_{{\boldsymbol{i}}+{\boldsymbol{j}}=1}^{2}{{\boldsymbol{C}}}_{{\boldsymbol{ij}}}{(\overline{{{\boldsymbol{I}}}_{1}}-3)}^{{\boldsymbol{i}}}{(\overline{{{\boldsymbol{I}}}_{2}}-3)}^{{\boldsymbol{j}}}+{\sum }_{{\boldsymbol{i}}=1}^{2}\frac{1}{{{\boldsymbol{D}}}_{{\boldsymbol{i}}}}{({{\boldsymbol{J}}}_{{\boldsymbol{el}}}-1)}^{2{\boldsymbol{i}}}$$

in which ***U*** is the strain energy per unit of reference volume; $\overline{{{\boldsymbol{I}}}_{1}}\,\,$and $\overline{{{\boldsymbol{I}}}_{2}}$ represent the first and second deviatoric strain invariant; ***J***_***el***_ is the elastic volume ration; ***C***_***ij***_ and ***D***_***i***_ are the input coefficients of hyperelasticity parameters, which were determined by a stress-strain curve obtained from *in vivo* test of the heel^55^. The bones and the encapsulated soft tissue were meshed with 4-node linear tetrahedral elements, plantar fascia and ligaments were meshed with 2-node linear 3-D truss elements, and the two layers of plates beneath the plantar foot were meshed with 8-node linear hexahedral elements.

FE models of the TAA foot and ankle arthrodesis foot were obtained through modifying the ankle joint in the intact foot model. A three-component ankle prosthesis (STAR, Scandinavian Total Ankle Replacement) was implanted to the model (Fig. 6) to simulate the TAA foot. This prosthesis consisted of a tibial plate, a talar component, and a mobile bearing, each of which were aligned in their corresponding positions to replace the ankle joint in the foot model following standard surgical procedures. The tibial plate and the talar component were tied to the cutting surface of the tibia bone and the talus bone, respectively, while the mobile bearing was inserted and allowed to slide between the tibial plate and the talar component. The contact property was set as frictionless “surface-to-surface”. The sizes of the tibial plate and talar component were extra small (30 mm × 30 mm) and extra-extra small (28 mm × 29 mm), respectively. The thickness of the mobile bearing was 6 mm. The Young’s modulus and Poisson’s ratio were set as 116000 MPa and 0.32, respectively, for the tibial plate and talar component, which were made of cobalt-chromium-molybdenum alloy. The mobile bearing was set as 810 MPa and 0.46 to represent the material properties of ultra-high molecular weight polyethylene. All three components were meshed into 4-node linear tetrahedral elements.

Ankle arthrodesis is a procedure that removes the cartilage on the articulation interface and fuses the talus and tibia bones together as one bone, without any correction intervention on other bones or soft tissues. This surgery can be implemented with various operative techniques regarding approaches (open or arthroscopic) and fixation methods (internal or external fixation), with all the technique having the same objective of completely constraining the ankle joint motion. To represent the surgery, the interaction pattern of the ankle joint in the FE model was changed from frictionless ‘surface-to-surface’ contact to ‘tie’ connection, which totally constrained the relative motion between the tibia and talus bones. All the other bony and soft tissue structures and the mesh and material property settings in the ankle arthrodesis were kept the same as the model of the intact foot.

The boundary and loading conditions for gait simulation were obtained from gait analysis on the same subject as for the intact foot model. The gait analysis was conducted in a biomechanical lab with a 10-meter walk distance equipped with a 3D motion capture system with eight cameras (Vicon, Oxford Metrics, Oxford, UK) and two force platforms (AMTI, Advanced Mechanical Technology, Inc., Watertown, MA, USA). To record the kinetic and kinematic information, 16 reflective markers were attached to the lower limbs of the subject, defining seven segments including the pelvis, two thighs, two lower legs, and two feet. A pressure measurement system (F-Scan, TekScan Inc., Boston, MA, USA) was used to measure the plantar pressure distribution during gait for model validation. Each sensor was trimmed to fit the foot size, and two sensors were attached to the plantar feet using double-sided adhesive tape.

**Figure 7:**
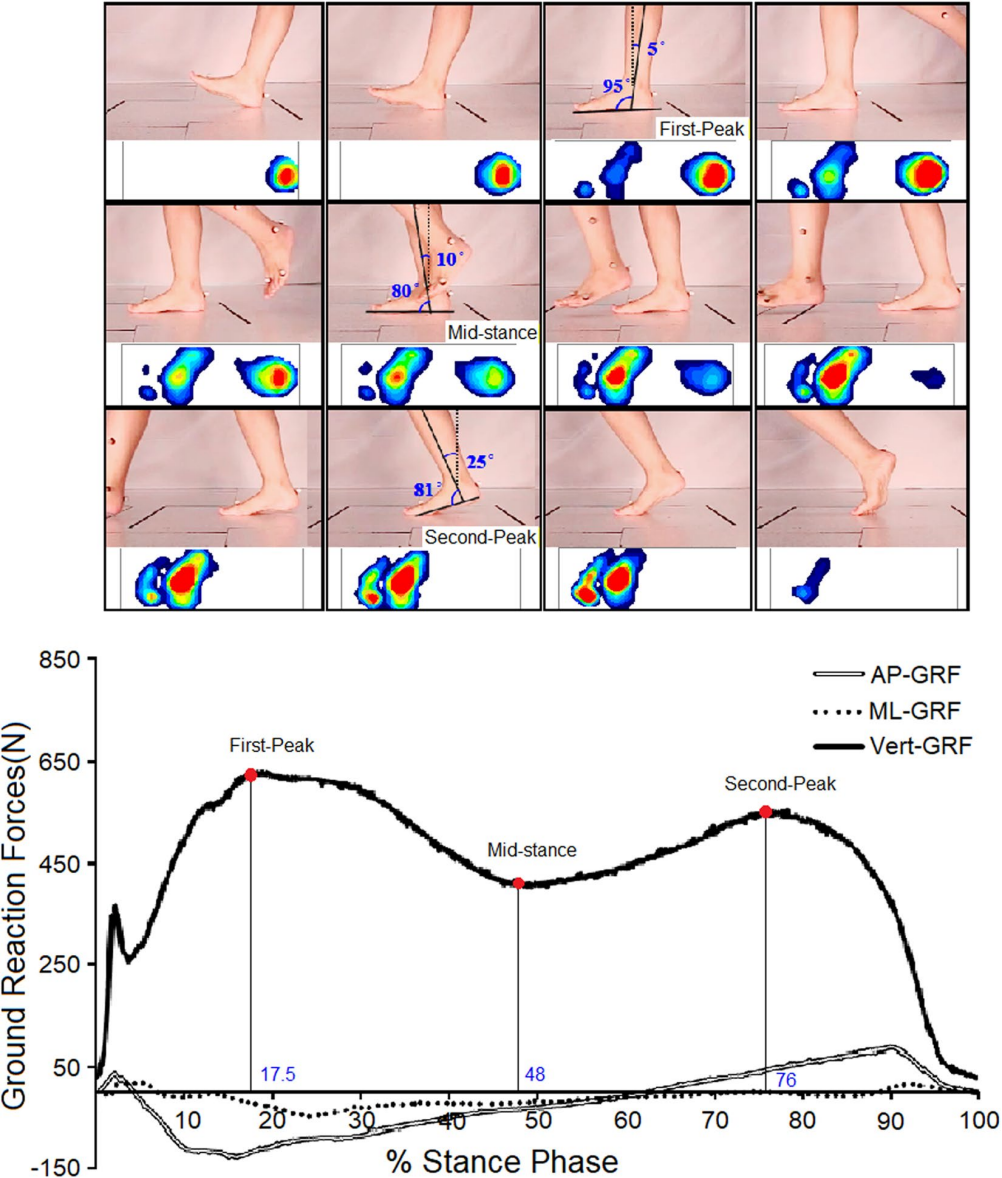
Foot positions, plantar pressure distribution, and curves of ground reaction forces during the stance phase of gait at the three marked instants, with AP-GRF, ML-GRF and Vert-GRF for anteroposterior, mediolateral and vertical ground reaction forces.

The subject was instructed to walk at her natural speed, with each foot stepping on a separate force platform. Ten trials of walking were conducted. In this procedure, the trajectories of the markers, ground reaction forces, and plantar pressure distribution of each foot were recorded. The shank-ground angle was calculated based on the marker trajectories in the lower limb. Ten trials of data were resampled with a uniform sample size, and then the averaged ground reaction force and shank-ground angle were obtained from the 10 trials. Figure 7 shows the curves of the ground reaction forces averaged from the 10 trials of natural-speed walking and plantar pressure distribution at three instants. Two peaks and a valley appeared in the curve of the vertical ground reaction force during the stance phase, with the first peak at 17.5% of the stance phase, the valley at 48%, and the second peak at 76% of the stance phase. The curves of the ground reaction forces and the three instants have been mentioned in the study on ankle arthrodesis^30^, and in the figure of this paper the positions of the foot and tibia and the plantar pressure distribution during the stance phase were involved. The three instants, named the first-peak, mid-stance, and second-peak instants, were simulated. The muscle forces ***F***_***i***_ were estimated from the physiological cross-sectional area (PCSA) of the muscles and normalized electromyography (EMG) data of barefoot walking assuming a linear EMG-force relationship (Eq. 2)^56^, in the form of

2 $${{\boldsymbol{F}}}_{{\boldsymbol{i}}}={{\boldsymbol{g}}}_{{\boldsymbol{i}}}\cdot {\boldsymbol{EM}}{{\boldsymbol{G}}}_{{\boldsymbol{i}}}\cdot {\boldsymbol{PCS}}{{\boldsymbol{A}}}_{{\boldsymbol{i}}}$$

with a constant muscle gain ***g***_***i***_ as 25 N/cm^2^ ^57^. The EMG data was adopted from the gait analysis study, which was normalized to eliminate the effects of individual differences^58^. The obtained shank-ground angle, ground reaction forces, and muscle forces at the three instants were applied as the boundary and loading conditions of the models.

The model of the intact foot was validated through comparison of the FE predictions and experimental measurements. One comparison was of the plantar pressure between the FE prediction and the F-Scan measurement in the gait experiment, and the other comparison was of the joint contact pressure between the FE prediction and the measurement in a cadaver experiment^30^.
